# Neural Temporal Dynamics of Facial Emotion Processing: Age Effects and Relationship to Cognitive Function

**DOI:** 10.3389/fpsyg.2017.01110

**Published:** 2017-06-30

**Authors:** Xiaoyan Liao, Kui Wang, Kai Lin, Raymond C. K. Chan, Xiaoyuan Zhang

**Affiliations:** ^1^Department of Psychology, School of Public Health, Southern Medical UniversityGuangzhou, China; ^2^Nanfang Hospital, Southern Medical UniversityGuangzhou, China; ^3^Neuropsychology and Applied Cognitive Neurosciences Laboratory, Institute of Psychology, Chinese Academy of SciencesBeijing, China; ^4^CAS Key Laboratory of Mental Health, Institute of Psychology, Chinese Academy of SciencesBeijing, China

**Keywords:** aging, cognitive function, event-related potential, N170, emotion

## Abstract

This study used event-related potentials (ERPs) to investigate the effects of age on neural temporal dynamics of processing task-relevant facial expressions and their relationship to cognitive functions. Negative (sad, afraid, angry, and disgusted), positive (happy), and neutral faces were presented to 30 older and 31 young participants who performed a facial emotion categorization task. Behavioral and ERP indices of facial emotion processing were analyzed. An enhanced N170 for negative faces, in addition to intact right-hemispheric N170 for positive faces, was observed in older adults relative to their younger counterparts. Moreover, older adults demonstrated an attenuated within-group N170 laterality effect for neutral faces, while younger adults showed the opposite pattern. Furthermore, older adults exhibited sustained temporo-occipital negativity deflection over the time range of 200–500 ms post-stimulus, while young adults showed posterior positivity and subsequent emotion-specific frontal negativity deflections. In older adults, decreased accuracy for labeling negative faces was positively correlated with Montreal Cognitive Assessment Scores, and accuracy for labeling neutral faces was negatively correlated with age. These findings suggest that older people may exert more effort in structural encoding for negative faces and there are different response patterns for the categorization of different facial emotions. Cognitive functioning may be related to facial emotion categorization deficits observed in older adults. This may not be attributable to positivity effects: it may represent a selective deficit for the processing of negative facial expressions in older adults.

## Introduction

Facial emotion processing is affected by aging (Di Domenico et al., 2015) and clinical conditions (Gur et al., 2007; Weiss et al., 2008; Wieser et al., 2012; Altamura et al., 2016). Numerous behavioral studies have identified age-related decline of labeling negative facial expressions and a preservation in labeling happy expressions (Isaacowitz et al., 2007; Ruffman et al., 2008; Horning et al., 2012; West et al., 2012; Kessels et al., 2014). Neuroimaging data have also found decline of neural activities to negative stimuli with age, and a relatively invariant response to positive stimuli across the adult life span (Gunning-Dixon et al., 2003; Tessitore et al., 2005; Fischer et al., 2010). It is argued that there is an attention (Mather and Carstensen, 2003) and memory (Charles et al., 2003) biases toward positive versus negative stimuli in older adults, so called the “positivity effect" (Mather, 2012; Nashiro et al., 2012). From a neurobiological perspective, the interaction between noradrenergic activity and emotional memory enhancement in older adults is considered relevant (Mammarella et al., 2016). This perspective has extended to the perception and identification of another’s expression (Bucks et al., 2008; Isaacowitz et al., 2009; Kaszniak and Menchola, 2012). A competing perspective for explaining age-related changes in labeling negative expressions has been argued as a general decline of cognitive function (Suzuki and Akiyama, 2013) or neurological atrophy in specific brain regions (Calder et al., 2003).

The processing of facial expression information is fast and efficient (Vuilleumier, 2005), and may lead to substantial temporal dispersion of evoked responses that enable ‘high-level’ regions to respond with surprisingly short latencies (Kawasaki et al., 2001; Pessoa and Adolphs, 2010). With resolution in the order of milliseconds, event-related potentials (ERP) are candidates to be excellent neural markers of the early involvement of perceptual face knowledge (Rossion, 2014). Affective facial stimuli elicit particular ERP components (Eimer and Holmes, 2007; Luo et al., 2010; Leleu et al., 2015), such as: (a) the P1, a positive potential with peak latency from 70 to 130 ms after stimulus onset over the occipital brain scalp sites, indicating selective spatial attention toward emotional cues (Luck et al., 2000; Bekhtereva et al., 2015); (b) the N170, a prominent negative waveform over the occipito-temporal scalp sites, with a peak at approximately 170 ms post-stimulus, representing an early neural marker involved in the pre-categorical structural encoding of an emotional face (Rossion, 2014); (c) the posterior P2, a positive deflection observed over the occipito-temporal regions at approximately 200–280 ms post-stimulus (Latinus and Taylor, 2006; Brenner et al., 2014), which has been suggested to be a kind of stimulus-driven call for processing resources (van Hooff et al., 2011); and (d) the N250, an affect-related negative component peaking at approximately 250 ms post-stimulus over the occipito-temporal scalp sites (Streit et al., 2000; Williams et al., 2006).

Event-related potential studies have used diverse paradigms to examine age-related changes of facial emotion processing, including passive viewing (Smith et al., 2005; Hilimire et al., 2014; Mienaltowski et al., 2015) and emotion recognition memory tasks (Schefter et al., 2012). Hilimire et al. (2014) suggest that there is an age-related shift away from negative faces toward positive faces within an early (110–130 ms) and late (225–350 ms) period in a checkerboard probe go/no-go task requiring passive processing of task-irrelevant emotional faces. However, another previous study of young adults showed that the processing of negative faces was increased at early perceptual stages only task-irrelevantly (e.g., passive viewing), whereas happy faces received enhanced processing only task-relevantly (e.g., naming the emotional expression) (Rellecke et al., 2012). To further test the effect of age on facial emotion processing, it would be necessary to manipulate the relevance to the task of the facial expressions. This would allow researchers to determine whether older adults demonstrate enhanced processing of positive expressions during explicit facial emotion identification tasks, and whether greater processing in response to positive faces is associated with stronger cognitive functioning.

The goal of the present study was to revisit the effect of age on particular ERP components using facial emotion categorization paradigm. To shed light on the controversy regarding the mechanism underlying these age effects, correlations between cognitive functions and brain responsivity to specific emotional faces, as well as the correlations between cognitive functions and performance of labeling facial emotions, were also explored. We used a reference sample of healthy younger adults to test the specificity of the effects in an older population. Given the existence of two directly competing perspectives discussed above, no specific directional hypothesis was possible. If available cognitive resources may be voluntarily engaged, accounting for the positivity effects observed in older adults (Isaacowitz et al., 2009), we hypothesized that higher cognitive abilities should be associated with reduced or enhanced neural responsivity to specific emotional faces. If impaired performance on labeling negative facial expressions is an unintended consequence of a general decline of cognitive function associated with old age (Suzuki and Akiyama, 2013), we anticipated that weaker cognitive abilities might show a correlation with decreased performance in labeling negative faces in older adults (but not young adults), but not necessarily in labeling positive faces.

## Materials and Methods

### Participants

Thirty healthy older adults (58–79 years of age; 17 female) and 31 young adults (22–26 years of age; 19 female) were recruited. All participants were right-handed and had normal or corrected-to-normal vision and normal hearing. A battery of neuropsychological tests, including the Montreal Cognitive Assessment (MoCA), the Auditory Verbal Learning Test (AVLT), the Logical Memory Test (LMT), and the forward and backward digit span test, were used to confirm that participants were within normal ranges for cognitive functioning according to published norms (Guo et al., 2009). Meanwhile, older adults completed the Instrumental Activities of Daily Living scale (IADL) and the Geriatric Depression Scale (GDS), while young adults completed the Center for Epidemiologic Studies Depression Scale (CES-D).

For both groups, inclusion criteria were the absence of self-reported history of neuropsychological impairment or any disorder affecting the central nervous system, no previous head injury, and not currently being treated for depression or anxiety. Exclusion criteria were (a) impaired general cognition and activities of daily living; (b) subjective memory impairment, and scoring more than 1.5 standard deviations below the age and education-adjusted mean on the immediate or delayed AVLT or LMT; and (c) a GDS score > 20 for older adults (Chan, 1996); and (d) a CES-D score > 28 for young adults (Cheng et al., 2006). Participants’ characteristics are shown in **Table 1**.

**Table 1:** Participants characteristics.

Items	Older adults (*n* = 30)		Young adults (*n* = 31)	
	Mean	*SD*	Mean	*SD*
Age (range) (years)	67.77 (58–79)	6.54	23.52 (22–25)^∗∗∗^	0.85
Sex (male, %)	13 (43%)	/	16 (52%)	/
Education (years)	12.83	2.72	16.74^∗∗∗^	0.44
MoCA scores	27.27	1.62	29.39^∗∗∗^	0.76
AVLT scores (immediate)	8.77	1.76	10.89^∗∗∗^	1.26
AVLT scores (delayed)	8.17	1.49	10.90^∗∗∗^	1.16
LMT scores (immediate)	10.05	2.29	12.32^∗∗∗^	1.48
LMT scores (delayed)	7.98	2.78	11.61^∗∗∗^	1.36
Longest digit forward scores	7.64	0.90	9.35^∗∗∗^	1.14
Longest digit backward scores	4.82	0.80	7.87^∗∗∗^	1.15
Semantic fluency scores	20.20	5.14	23.26^∗^	4.17
IADL scores	12.93	1.74	/	/
GDS scores	6.73	3.91	/	/
CES-D scores	/	/	8.97	6.2

*MoCA, the Montreal Cognitive Assessment; AVLT, the Auditory Verbal Learning Test; LMT, the Logical Memory Subtests; IADL, the Instrumental Activities of Daily Living Scale; GDS, Geriatric Depression Scale; CES-D, Center for Epidemiologic Studies Depression Scale. SD, standard deviation; ^∗^*p* < 0.05; ^∗∗∗^*p* < 0.001*.

Participants were recruited from the Southern Medical University (Guangzhou, China) and the nearby community via advertisements, and received monetary compensation. All participants provided written informed consent in accordance with the Declaration of Helsinki. The study was approved by the Medical Ethics Committee of Nanfang Hospital of Southern Medical University (NFEC-201511-K2).

### Stimuli and Task

Ninety-six digitally reworked black-and-white face photographs were selected from the Chinese Facial Affective Picture System database (Luo et al., 2010). The faces expressed positive (happiness, 32 pictures), neutral (32 pictures), and negative (32 pictures; fear, anger, sadness and disgust, eight pictures each) emotions, and were balanced for gender and matched for luminance and contrast grade. Only closed-mouth expressions were used. Positive and negative faces were equated in terms of emotional intensity (*t* = -1.34, *p* = 0.187). Mean emotional intensity was 5.85 (SEM = 0.27) and 6.29 (SEM = 0.18) for positive and negative faces. Each face was seen by each participant once. The pictures used in the practice stage were different from those used in the formal experiment.

The stimuli were presented in random order for 500 ms each, against a black background subtending a visual angle of about 11° to 15° at a distance of 75 cm. Participants were instructed to classify the displayed facial expressions from a multiple-choice scale containing the names of the six facial expressions as discrete categories arranged horizontally on the screen. The scale appeared on the screen after a delay of 1000 ms (to avoid confounding motor effects), and was presented until a classification had been made, or for a maximum of 8000 ms. A random interval of 1600–2200 ms was set between the participants’ response and the onset of the next trial. Emotions were classified by clicking the corresponding label of the scale with a computer mouse. The task paradigm has been described in a previous study (Woelwer et al., 2012).

### Event-Related Potentials

EEG data were collected from 32 electrodes (impedance < 5 kΩ), in accordance with the extended 10/20 system. Recordings were made with a Brain-Amp-DC amplifier and controlled through Brain Vision Recorder 2.0 (sampling rate: 250 Hz; recording reference: left mastoid) (Brain Products, Munich, Germany). Data were analyzed by Brain Vision Analyzer 2.0 (Brain Products) and filtered offline (band-pass 0.1–70 Hz with a 50-Hz notch filter; re-calculated to Cz reference), corrected for horizontal and vertical ocular artifacts, and baseline corrected to 200 ms pre-stimulus. Trials with a transition threshold of 50 μV (sample to sample) or an amplitude criterion of more than ±80 μV were automatically rejected.

The procedure was confirmed by visually checking the remaining trials for artifacts. The number of artifact-free trials did not differ between groups [(*F*_1,59_ = 0.81, *p* = 0.78), mean number of artifact-free trials per valence: positive faces = 31.97 (*SD* = 0.18), neutral faces = 31.97 (*SD* = 0.18), and negative faces = 31.93 (*SD* = 0.37) in older adults; positive faces = 31.94 (*SD* = 0.36), neutral faces = 31.90 (*SD* = 0.54), and negative faces = 32.19 (*SD* = 0.18) in young adults].

The amplitudes (measured as peak-to-baseline values) of the P100 (70–130 ms) and the N170 (130–200 ms) were averaged from occipital (O1 and O2) and parietal (averaged across P3/P7 and P4/P8, respectively) scalp sites, respectively. The time-windows were chosen by visually inspecting the time course of each component. Peaks of the components were measured within a ±30 ms window centered on the maximum of the grand-average means (Itier and Taylor, 2004). To further test whether there is a positive deflection to task-relevant happy faces in older adults within the time-window of P100, the ERP data were also re-calculated according to the average reference (average of all scalp electrodes), and reanalyzed using the method reported by Hilimire et al. (2014).

It was difficult to quantitatively compare the P2 and N250 components between the two age groups, because the older adults demonstrated a clearly delayed P2 (older adults: 270–330 ms; young adults: 200–260 ms) and near-absent N250 (or that which was merged into the later component). Therefore, we qualitatively compared the between-groups differences in brain responsivity after the N170 based on the topographical maps corresponding to the duration from 200 to 500 ms post-stimulus. Multiple re-entrant feedback signals along various regions of the visual ventral stream are thought to be surprisingly fast, with each processing stage adding approximately 10 ms to the overall latency (Pessoa and Adolphs, 2010). As the shortest possible time bin for the present topographical maps was 16 ms, the spline interpolated topographical maps of scalp voltage across 32 electrodes over the period from 200 to 500 ms post-stimulus were computed in consecutive 16-ms bins.

### Statistical Analyses

Event-related potentials were analyzed separately for signal amplitude at the corresponding electrodes and entered into repeated-measures ANOVAs with the Greenhouse-Geisser epsilon correction in case of violation of sphericity. The between-participant variable was “Group” (older adults vs. young adults), and the within-group variables were “Hemisphere” (two levels: left and right) and “Emotion” (three levels: positive, neutral, and negative). In case of a significant interaction including Group and Emotion, *post hoc* tests were calculated using Bonferroni correction to account for multiple testing. Independent-sample *t*-tests and chi-square tests assessed differences in demographic variables between the groups.

The multiple linear regression analyses (forward procedure) were performed to statistically test the correlations between cognitive functions and ERPs, as well as the correlations between cognitive functions and performances, for both groups. In the first regression analysis, amplitudes of P100 and N170 were introduced as the dependent variable, respectively. Scores of MoCA, AVLT, LMT, forward and backward digit span, semantic fluency, and age were introduced as the independent variables. In the second regression analysis, performance data (accuracy rates for negative, positive, and neutral faces) were introduced as dependent variable, respectively. Independent variables were the same as in the previous analysis.

## Results

### Behavioral Results

Regarding accuracy, a repeated-measure ANOVA yielded a Group × Emotion interaction (*F*_2,118_ = 11.32, *P* < 0.001, $\eta_{p}^{2}$ = 0.16). The older adults performed less accurately in labeling negative faces than young adults (*t* = -6.20, *P* < 0.001), while their performance for positive and neutral faces remained no significant group difference (*P* > 0.10). Within-group accuracy for labeling negative faces were significantly lower than those for labeling positive faces (older adults: *t* = -10.59, *P* < 0.001; young adults: *t* = -8.25, *P* < 0.001) and neutral faces (older adults: *t* = -8.87, *P* < 0.001; young adults: *t* = -6.84, *P* < 0.001) in both groups.

Reaction time mirrored the pattern of accuracy in the Group × Emotion interaction (*F*_2,118_ = 9.47, *p* = 0.001, $\eta_{p}^{2}$ = 0.14). After adjusting for reaction time in the practice phase before the formal experiment, older adults were slower to label negative faces (*F*_1,58_ = 9.92, *p* = 0.003, $\eta_{p}^{2}$ = 0.15) relative to young adults, while their performance for labeling positive and neutral faces remained no significant difference (*p* > 0.10). Detailed behavioral results are reported in **Table 2**.

**Table 2:** Between-group differences in performance of facial emotion categorization task.

Variables	Older adults (*n* = 30)		Young adults (*n* = 31)		*t*	*p*	*t*^a^	*p*^a^
	Mean	*SD*	Mean	*SD*				
**ACC (%)**								
Positive	85	-13	89	-8	-1.53	0.131		
Neutral	86	-16	92	-10	-1.52	0.134		
Negative	56	-14	75	-10	-6.20	0.000		
Practice phase	93	-8	95	-6	-1.67	0.167		
**RT (milliseconds)**								
Positive	849.11	-323.52	686.07	-280.88	2.51	0.036	1.61	0.113
Neutral	847.22	-375.89	668.09	-267.06	2.10	0.040	1.59	0.118
Negative	1726.09	-547.90	1221.44	-368.57	4.23	0.000	1.61	0.003
Practice phase	1539.50	-741.43	891.12	-339.53	4.81	0.000		

*Abbreviations: RT, reaction time; ACC, accuracy rate; MD, mean difference; SD, standard deviation; ^a^Yielded from one-way ANOVA adjusted by reaction time in the practice phase*.

### Electrophysiological Results

As for P100 amplitudes, none of the interactions that included Group × Emotion were statistically significant (*F* < 1). Descriptive statistics for ERP variables are in Supplementary Tables S1, S2.

As for N170 amplitudes, a significant Group × Hemisphere × Emotion interaction was observed (*F*_2,118_ = 3.08, *P* = 0.05, $\eta_{p}^{2}$ = 0.05). Group differences were detected for left hemisphere (negative faces: *t* = -3.31, *P* = 0.002; positive faces: *t* = -3.29, *P* = 0.002; and neutral faces: *t* = -4.00, *P* < 0.001), and right hemisphere (negative faces: *t* = -2.24, *P* = 0.003; and neutral faces: *t* = -2.12, *P* = 0.04). No group difference was found for right-hemispheric N170 amplitudes elicited by positive faces (*P* > 0.10).

**FIGURE 1:**
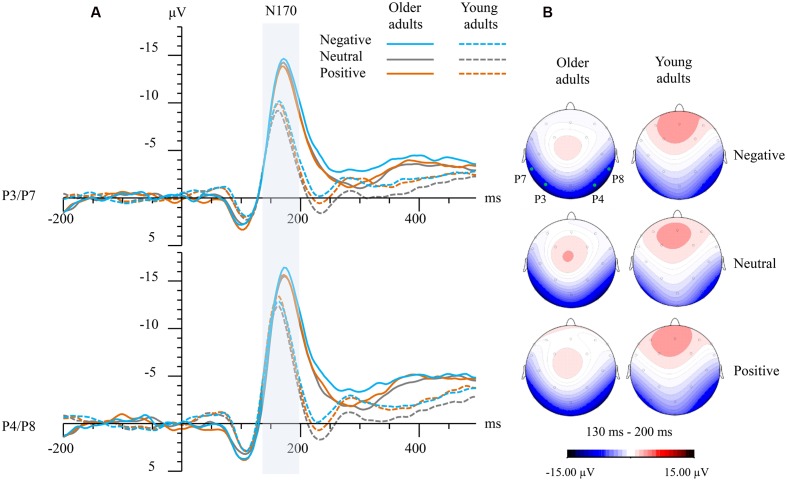
**(A)** Grand-averaged event-related potential (ERP) waveforms elicited at left (averaged from P3 and P7) and right temporo-occipital electrodes (averaged from P4 and P8) for both groups and emotions. The vertical bar marks the time-windows that were analyzed as N170 in both age groups. **(B)** Topographical maps showing top view of scalp distributions over the N170 time-window for negative faces (top), neutral faces (medium), and positive faces (bottom). Note: the solid green points indicate the scalp electrodes where the P3, P4, P7, and P8 were located.

Bonferroni corrected *post hoc* tests showed that there was no significant within-group emotion effect in older adults. In contrast, young adults demonstrated that the N170 elicited by emotional faces were larger than those elicited by neutral faces (negative vs. neutral: *t* = -2.99, *P* = 0.02; positive vs. neutral: *t* = -2.90, *P* = 0.02) at the left hemisphere, and N170 elicited by positive faces was larger than those elicited by neutral faces (*t* = -2.94, *P* = 0.02) at the right hemisphere. Moreover, within-group analysis in older adults showed that the right-hemispheric N170 was significant larger than left-hemispheric N170 for emotional faces (negative faces: *t* = -2.34, *P* = 0.026; positive faces: *t* = -2.61, *P* = 0.014), while the N170 laterality effect was not significant for neutral faces (*P* = 0.07). On the contrary, the N170 laterality effect has observed for all emotions in young adults (negative faces: *t* = -5.93, *p* < 0.001; neutral faces: *t* = -5.54, *P* < 0.001; positive faces: *t* = -4.69, *P* < 0.001). See also **Figure 1**.

**FIGURE 2:**
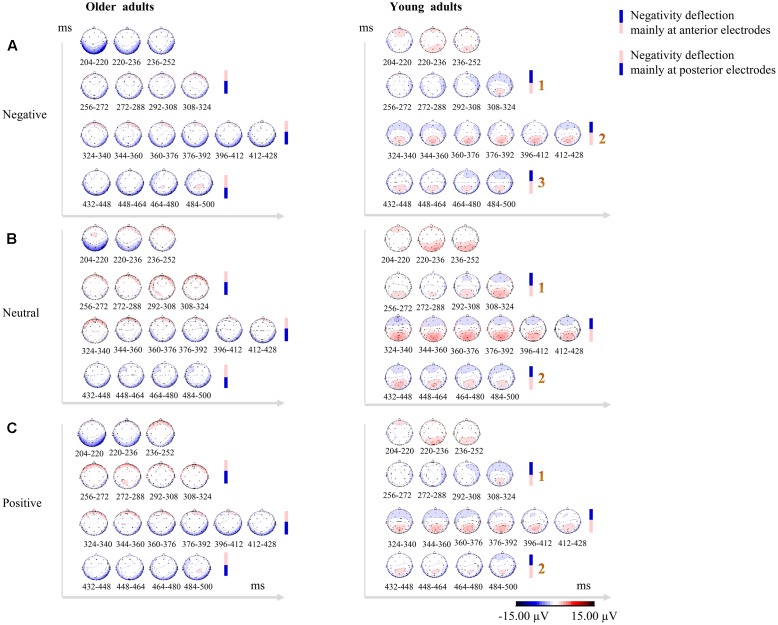
Topographical maps in consecutive 16-ms bins over the time range of 200–500 ms post-stimulus showing qualitative between-group differences in scalp distributions across 32 electrodes for negative faces **(A)**, neutral faces **(B)**, and positive faces **(C)**. Older adults exhibited sustained temporo-occipital negativity deflections (mainly at posterior electrodes), while young adults showed clear posterior positivity over P2 time-window and subsequent frontal negativity deflections (mainly at anterior electrodes). The negativity deflections in young adults were thrice enhanced (scalp voltage gradually increasing thrice) for negative faces, and twice enhanced (scalp voltage gradually increasing twice) for neutral and positive faces. Enhanced positivity for the stimuli is shown in red, while blue indicates enhanced negativity for the stimuli. The scale (–15/+15 μV) has been adapted to better display the topographical similarities and differences.

Older adults exhibited a sustained temporo-occipital negativity deflection (mainly at posterior electrodes) over the time range of 200–500 ms post-stimulus. By contrast, young adults showed clear posterior positivity and subsequent frontal negativity deflection (mainly at anterior electrodes). Moreover, the frontal negativity deflections observed in young adults were thrice enhanced (scalp voltage gradually increasing thrice) for negative faces and twice enhanced (scalp voltage gradually increasing twice) for neutral and positive faces between 256 and 500 ms post-stimulus (see **Figure 2**).

### Correlations between Cognitive Functions, Task Performance, and ERPs

**FIGURE 3:**
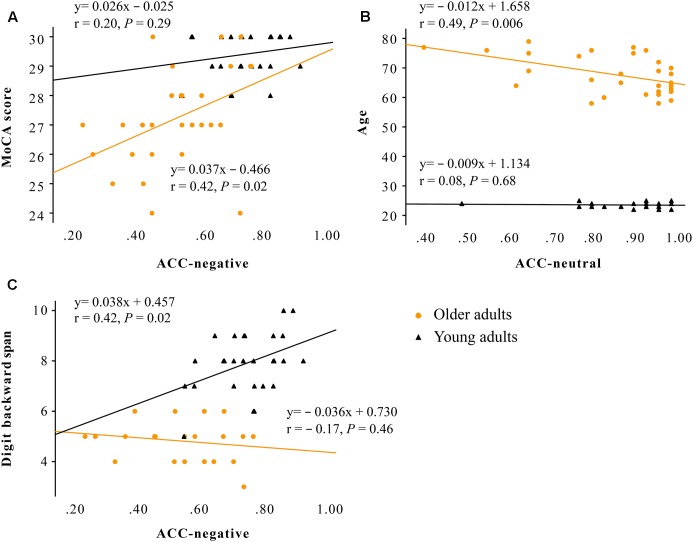
Scatter plot showing that: **(A)** decreased accuracy for labeling negative faces was correlated with lower MoCA scores only in older adults (adjusted *R*^2^ = 0.15, standardized Beta = 0.42, *P* = 0.020); **(B)** decreased accuracy for labeling neutral faces was correlated with advanced age only in older adults (adjusted *R*^2^ = 0.21, standardized Beta = -0.49, *P* = 0.006); **(C)** decreased accuracy for negative faces was correlated with lower backward digit span only in young adults (adjusted *R*^2^ = 0.15, standardized Beta = 0.42, *P* = 0.019). Older adults are marked with circles, and younger adults are marked with triangles. Note: ACC, accuracy rate; MoCA, the Montreal Cognitive Assessment.

No correlations were observed between cognitive functions (scores of MoCA, AVLT, LMT, forward and backward digit span, semantic fluency) and ERPs (amplitudes of P100 and N170) in either group (*P* > 0.05). For the correlations between cognitive functions and task performances (expressed as accuracy for labeling negative, positive, and neutral faces) in older adults, decreased accuracy for labeling negative faces was correlated with lower MoCA scores (adjusted *R*^2^ = 0.15, standardized Beta = 0.42, *P* = 0.020; **Figure 3A**), and decreased accuracy for labeling neutral faces was correlated with advanced age (adjusted *R*^2^ = 0.21, standardized Beta = -0.49, *P* = 0.006; **Figure 3B**). In young adults, decreased accuracy for labeling negative faces was correlated with lower backward digit span (adjusted *R*^2^ = 0.15, standardized Beta = 0.42, *P* = 0.019; **Figure 3C**).

## Discussion

### Facial Emotion Labeling Performance

A large sample internet-based explicit emotion identification study suggests an inverted U-shaped trajectory over 6–91 years with highest identification accuracy in the middle decades (20–49 years) and a progressive decline over 50–91 years (Williams et al., 2009). The behavioral results in the present study are highly similar to this kind of trajectory (Ruffman et al., 2008; Kessels et al., 2014), wherein reduced accuracy and slower speed for identification of negative faces and relatively preserved identification of positive and neutral faces are observed in older adults. It is possible that emotion labeling of negative facial emotions is more difficult than that of positive and neutral faces. In contrast, happy faces are easier to recognize because other positive emotions are absent as possible distractors (Kessels et al., 2014), and a smile may be a shortcut for quick and accurate categorization (Beltran and Calvo, 2015).

### Event-Related Potentials

The P100 has previously been associated with attention-related enhancements of processing intrinsically salient stimuli (Di Russo et al., 2003). In the present study, an insignificant group difference in P100 amplitudes suggests that early detection of facial emotions remains relatively intact in the older adults. We did not observe an attention bias toward positive or away from negative faces over the P100 time-window in the present study. A recent ERP study reported an age-related stronger positive deflection for task-irrelevant happy faces at frontal scalp sites over the time-window of P100, when performing a checkerboard probe go/no-go task (Hilimire et al., 2014). We also re-calculated the ERP data using the method reported by Hilimire et al. (2014), but no positive deflection was observed. One of the potential explanations for this inconsistence is that task relevance may manipulate the effect of on facial emotion processing.

The N170 component has excellent test-retest reliability, and obtaining as few as 10 or 20 artifact-free trials per condition for each participant may be sufficient to attain adequate reliability (Huffmeijer et al., 2014). The N170 is linked to pre-categorical structural encoding of emotional faces, where generated holistic internal face representations are used by subsequent finer-grained processing of expression categorization (Calvo and Beltran, 2014; Rossion, 2014; Bekhtereva et al., 2015). In line with our behavioral results, the present study revealed that the N170 amplitudes elicited by negative faces were enhanced in older adults relative to their younger counterparts, while the group difference in right-hemispheric N170 elicited by positive faces was non-significant, reflecting that older adults may exert more effort in structural encoding of facial features in negative faces, while their structural encoding of positive faces may be relatively preserved. Recent visual evoked potential studies suggest that emotional cue extraction for faces might be completed within the N170 phase (Bekhtereva et al., 2015), and N170 amplitudes could distinguish emotional faces from neutral faces in younger adults (Zhang et al., 2013). Accordingly, compromised within-group emotion effect on N170 amplitudes observed in our older adult participants may go some way to explaining the decreased differentiation of emotional faces from neutral faces in older adults.

Interestingly, the present study also observed decreased hemispheric laterality effect for the N170 elicited by neutral faces in older adults. In contrast, young adults exhibited an obvious N170 laterality effect for all facial emotions. It has been suggested that facial expression processing is both holistic and analytic (Tanaka et al., 2012). Holistic processing is thought to be preferentially executed by the right hemisphere, whereas the left hemisphere is regarded as more involved in part-based processing (Ramon and Rossion, 2012). The left-hemispheric N170 was previously found to be greater for featural relative to configural changes (Calvo and Beltran, 2014), whereas the right-hemispheric N170 shows the opposite pattern (Scott, 2006). Therefore, the non-significant group difference in right-hemispheric N170 for positive faces observed in the present study may suggest that the holistic processing of positive faces remains relatively intact in the older adults, while attenuated N170 laterality effect for neutral faces may reflect compensatory engagement of additional regions during pre-categorical structural encoding of facial emotions in older adults.

As a result of the obvious phase delay of the P2, and near-absent N250 in older adults, it was difficult to quantitatively compare the P2 and the N250 between two age groups. By using the topographical maps in consecutive 16-ms bins over the time range of 200–500 ms post-stimulus to qualitatively compare between-group difference in scalp distributions for three valence conditions, the present study revealed a sustained temporo-occipital negativity deflection in older adults and frontal negativity deflections in young adults over this time range. Moreover, the frontal negativity deflections in young adults were thrice enhanced in response to negative faces and twice enhanced in response to neutral and positive faces. These findings suggest that two age groups may have recruited different frontal-parietal networks during the facial emotion labeling task, and exert different strategies for processing facial expressions.

### Correlations of Cognitive Functions, Task Performance, and ERPs

Prefrontal and temporal-lobe structures that are important in recognizing and naming facial emotional stimuli in general (Kober et al., 2008; Ruffman et al., 2008) were found to be the earliest and most strongly affected by advancing age (Petit-Taboue et al., 1998). These brain regions are implicated in conceptualization (categorical perception of discrete emotions), language (representation of feature-based information for abstract categories), and executive attention (volitional attention and working memory), suggesting that more “cognitive” functions play a routine role in constructing perceptions of facial emotions (Lindquist and Barrett, 2012), especially when one explicitly evaluates and holds affective information in mind to categorize it.

In line with it, the present study found that older adults’ impaired performance for labeling negative faces was associated with decreased MoCA scores, suggesting that cognitive functions may contribute to impaired identification of negative faces in older adults. Moreover, older adults’ performance for labeling neutral faces was found to be negatively associated with age. This may partially explain the decreased N170 laterality effect for neutral faces observed in our older adult participants. Furthermore, cognitive functioning was not correlated with any early neural responses to emotional faces, suggesting that older adults may not voluntarily or involuntarily employ cognitive resources to modulate emotion perception during early visual processing of task-relevant emotional faces.

### Limitations

Limitations of this study should be considered and verified. First, we combined total artifact-free trials into the ERP analysis, instead of just taking correct trials, because of the significant group difference in accuracy for the task. The **Supplementary Figure S1** showed that the waveform of the correct trials in older adults was overlap with that of the total trials, but slightly higher than that of the incorrect trials. The combination may allow better understanding of actual early processing of a valence-specific emotion in older adults. Second, the pictures used in the present study were mainly younger faces, which may introduce the concern of “own-age bias” in face processing (Ebner et al., 2011). However, the processing of negative faces has also been reported to override age-of-face effects in facial expression identification tasks (Ebner et al., 2013). Further studies should be designed to use stimuli depicting both young and older adults.

## Conclusion

The present findings showed that older people may exert more effort during structural encoding and different response patterns during emotional decoding of facial expressions, especially for negative faces. Therefore, aging might be associated with a selective deficit in processing negative facial expressions. Further investigations in this area may facilitate the detection of at-risk individuals in the early stage of mild cognitive impairment and enable targeted early interventions.

## Author Contributions

XL designed the study, collected and analyzed the data, and drafted the manuscript. KW contributed to the experiment design and data interpretation. KL participated in the participants’ enrollment and cognitive assessment. XZ and RC conceived of and designed the study, and reviewed the manuscript. All authors have read and approved the final manuscript.

## Conflict of Interest Statement

The authors declare that the research was conducted in the absence of any commercial or financial relationships that could be construed as a potential conflict of interest.

## Supplementary Material

FIGURE S1 The supplementary figure showing that the waveform of the correct trials was overlap with that of the total trials, but slightly higher than that of the incorrect trials.
