# Supplementary figures and images for: Neural Temporal Dynamics of Facial Emotion Processing: Age Effects and Relationship to Cognitive Function
Source: Front Psychol. 2017 Jun 30;8:1110. doi: 10.3389/fpsyg.2017.01110 (PMC5492800; doi:10.3389/fpsyg.2017.01110)

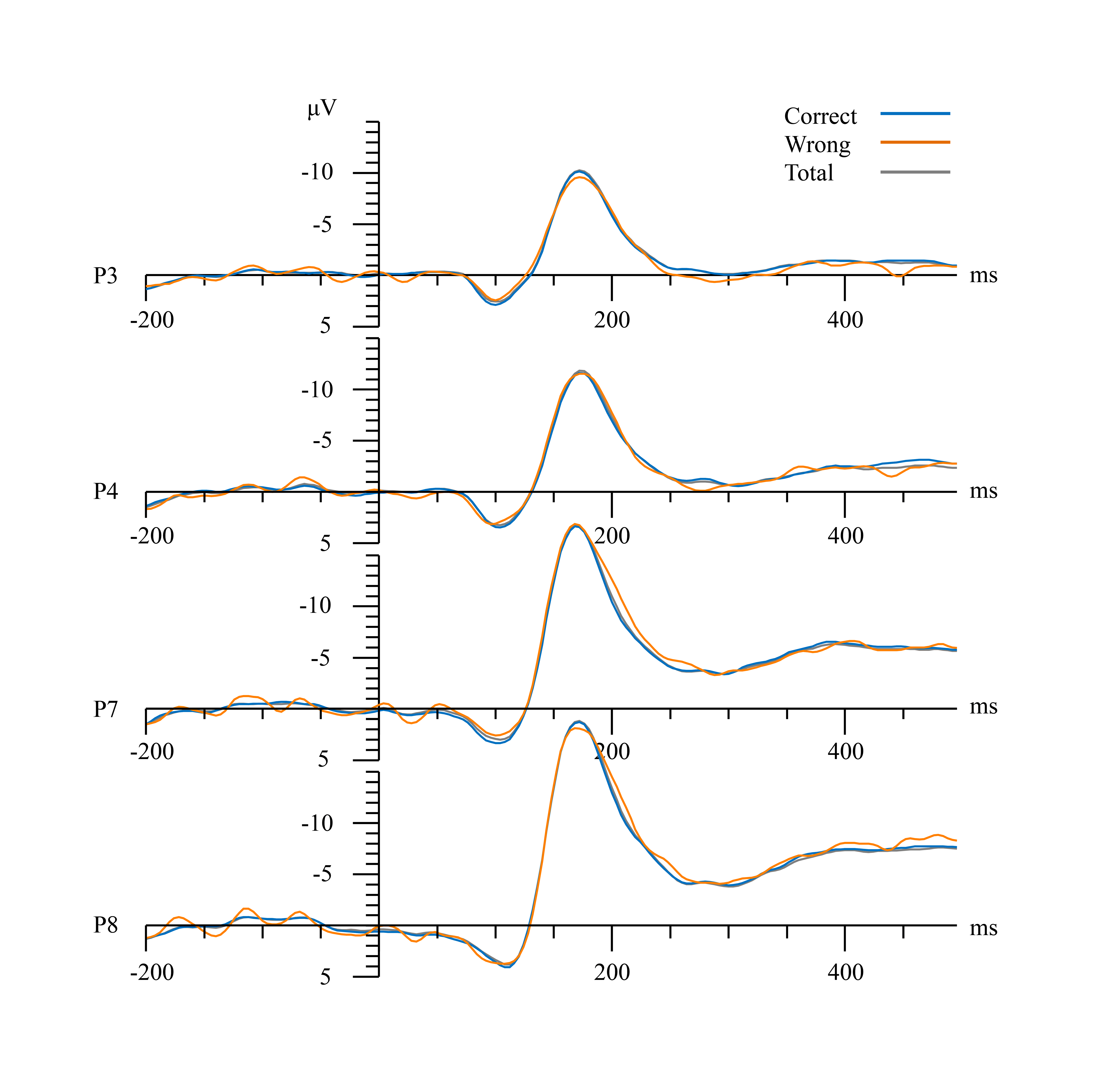

Supplement: FIGURE S1 — The supplementary figure showing that the waveform of the correct trials was overlap with that of the total trials, but slightly higher than that of the incorrect trials. [file Image_1.TIF]
