# Supplementary material for: Neural Temporal Dynamics of Facial Emotion Processing: Age Effects and Relationship to Cognitive Function
Source: Front Psychol. 2017 Jun 30;8:1110. doi: 10.3389/fpsyg.2017.01110 (PMC5492800; doi:10.3389/fpsyg.2017.01110)
Supplement: Supplementary file 2 [file Table_1.DOCX]

Table S1. Descriptive Statistics of Cz referenced ERP amplitudes (μVolt).

| Component | Electrode | Valence | Older adults (n=30) | |  | Young adults (n=31) | |
| --- | --- | --- | --- | --- | --- | --- | --- |
|  |  |  | Mean | SD |  | Mean | SD |
| P100 | O1 | negative | 5.97 | 3.73 |  | 5.09 | 4.14 |
|  |  | positive | 5.94 | 3.53 |  | 5.19 | 3.84 |
|  |  | neutral | 6.41 | 3.07 |  | 5.30 | 4.01 |
|  | O2 | negative | 6.33 | 3.61 |  | 5.68 | 3.97 |
|  |  | positive | 6.64 | 3.54 |  | 5.62 | 3.46 |
|  |  | neutral | 6.63 | 3.45 |  | 5.64 | 3.74 |
| N170 | P7 | negative | -20.16 | 8.37 |  | -14.00 | 5.70 |
|  |  | positive | -19.77 | 8.50 |  | -13.63 | 5.85 |
|  |  | neutral | -19.54 | 7.56 |  | -12.71 | 5.52 |
|  | P8 | negative | -22.63 | 10.42 |  | -17.32 | 5.64 |
|  |  | positive | -22.25 | 10.55 |  | -17.78 | 5.92 |
|  |  | neutral | -21.25 | 8.83 |  | -16.76 | 5.45 |
|  | P3 | negative | -11.56 | 4.99 |  | -8.43 | 5.01 |
|  |  | positive | -10.90 | 4.88 |  | -8.16 | 4.65 |
|  |  | neutral | -11.57 | 5.06 |  | -7.41 | 4.93 |
|  | P4 | negative | -13.08 | 6.34 |  | -10.81 | 5.13 |
|  |  | positive | -12.44 | 6.51 |  | -11.24 | 4.99 |
|  |  | neutral | -12.44 | 6.39 |  | -10.17 | 5.18 |
