# Supplementary material for: Neural Temporal Dynamics of Facial Emotion Processing: Age Effects and Relationship to Cognitive Function
Source: Front Psychol. 2017 Jun 30;8:1110. doi: 10.3389/fpsyg.2017.01110 (PMC5492800; doi:10.3389/fpsyg.2017.01110)
Supplement: Supplementary file 3 [file Table_2.DOCX]

Table S2. Descriptive Statistics of average referenced ERP amplitudes (μVolt).

| Component | Electrode | Difference score | Older adults (n=30) | |  | Young adults (n=31) | |
| --- | --- | --- | --- | --- | --- | --- | --- |
|  |  |  | Mean | SD |  | Mean | SD |
| P100 | F3 | Negative-Neutral | 0.04 | 1.61 |  | -0.05 | 1.41 |
|  |  | Positive-Neutral | 0.54 | 2.14 |  | -0.22 | 1.61 |
|  | F4 | Negative-Neutral | 0.18 | 1.54 |  | -0.05 | 1.67 |
|  |  | Positive-Neutral | 0.25 | 1.50 |  | -0.08 | 1.63 |
|  | Fz | Negative-Neutral | -0.14 | 1.30 |  | -0.26 | 1.69 |
|  |  | Positive-Neutral | 0.07 | 1.60 |  | -0.40 | 1.85 |
|  | C3 | Negative-Neutral | -0.17 | 1.33 |  | -0.20 | 1.16 |
|  |  | Positive-Neutral | -0.39 | 1.43 |  | -0.38 | 0.79 |
|  | C4 | Negative-Neutral | 0.09 | 1.42 |  | 0.04 | 1.16 |
|  |  | Positive-Neutral | 0.20 | 1.28 |  | 0.07 | 1.12 |
|  | Cz | Negative-Neutral | -0.13 | 0.99 |  | -0.17 | 1.29 |
|  |  | Positive-Neutral | -0.11 | 1.19 |  | -0.22 | 1.09 |
| N1 | P3 | Negative-Neutral | 0.07 | 1.33 |  | -0.21 | 0.99 |
|  |  | Positive-Neutral | 0.31 | 1.34 |  | -0.24 | 1.40 |
|  | P4 | Negative-Neutral | 0.53 | 2.08 |  | -0.05 | 1.17 |
|  |  | Positive-Neutral | 0.13 | 1.48 |  | 0.07 | 1.14 |
|  | Pz | Negative-Neutral | -0.14 | 0.95 |  | -0.69 | 0.95 |
|  |  | Positive-Neutral | -0.06 | 1.15 |  | -0.43 | 1.22 |
|  | O3 | Negative-Neutral | 0.34 | 2.49 |  | -0.23 | 2.00 |
|  |  | Positive-Neutral | 0.22 | 1.24 |  | -0.01 | 1.51 |
|  | O4 | Negative-Neutral | -0.06 | 1.11 |  | 0.12 | 1.81 |
|  |  | Positive-Neutral | -0.23 | 1.41 |  | 0.16 | 1.41 |
|  | Oz | Negative-Neutral | 0.13 | 1.46 |  | -0.33 | 1.57 |
|  |  | Positive-Neutral | 0.28 | 1.56 |  | -0.25 | 1.25 |

Note: These difference scores were obtained by subtracting the activity elicited by neutral faces from the activity elicited by the emotional expressions for each age group separately (Hilimire et al., 2014).
